# Multiband mucosectomy versus endoscopic submucosal dissection and endoscopic submucosal excavation for GI submucosal tumors: short and long term follow-up

**DOI:** 10.1186/s12885-019-6100-8

**Published:** 2019-09-06

**Authors:** Xi-Feng Jin, Wei Gai, Rong-Lian Du, Tong-Hai Chai, Ling Li, Christoph J. Auernhammer

**Affiliations:** 1 Department of Gastroenterology, TengZhou Central People’s Hospital, 183 Xingtan Road, Tengzhou, 277500 Shandong Province China; 2 Department of Internal Medicine 4, University-Hospital Campus Grosshadern, Ludwig-Maximilians-University of Munich, Munich, Germany

**Keywords:** Multiband mucosectomy (MBM), Endoscopic submucosal dissection (ESD), Endoscopic submucosal excavation (ESE), Submucosal tumours (SMTs), Gastrointestinal (GI)

## Abstract

**Aims:**

To evaluate the short- and long-term outcomes of 3 different endoscopic dissection techniques for upper gastrointestinal (GI) submucosal tumours (SMTs).

**Methods:**

Data for 135 patients withGI SMTs who underwent multiband mucosectomy (MBM), endoscopic submucosal dissection (ESD), or endoscopic submucosal excavation (ESE) were retrospectively assessed. The en bloc resection rate, endoscopic complete resection rate, operation time, potential complications and local recurrence rate were compared.

**Results:**

No significant differences were observed in the rate of endoscopic complete resections and pathologic complete resections among the three groups. For SMTs > 15 mm in width, the lowest en bloc resection rate was found for MBM (*P* = 0.000). MBM was also associated with the shortest procedure time, lowest perforation rate and lowest rate of major bleeding. ESE was the most effective procedure for muscularis propria (MP) lesions but was associated with the longest operation time (*P* < 0.01). The ESD and ESE groups had similar perforation rates (*P* > 0.05).

No differences were observed in 4-year local recurrence rates among the groups (*P* = 0.945).

**Conclusions:**

MBM is a simple and effective method for the treatment of small SMTs and achieves clinical success rates similar to those of ESD and ESE. However, ESD and ESE are preferable for larger and deep lesions and are associated with a longer operation time.

Nonetheless, all 3 techniques resulted in a low 4-year local recurrence rate. Large-scale randomized clinical trials are needed to further investigate these results.

## Background

Submucosal tumours (SMTs) are increasingly found incidentally during routine upper gastrointestinal (GI) endoscopies. Most SMTs are asymptomatic and therefore are clinically insignificant. However, SMTs exhibit potential for malignancy, particularly those originating from the muscularis propria (MP) layer. Therefore, resection is imperative.

Although open surgery is the standard treatment of choice, surgery has substantial rates of morbidity and mortality and seriously reduces the quality of life of SMT patients. Laparoscopic resection (LAP) is widely used for gastrointestinal stromal tumours (GISTs); this technique is beneficial to the patient because it is less invasive than open surgery [1–3]. However, over-resection or inadequate resection may result in dumping syndrome, severe reflux and GI stasis.

Endoscopic submucosal dissection (ESD) and endoscopic submucosal excavation (ESE) techniques have been described recently [4–8]. ESD has been used to resect both mucosal lesions and submucosal GI tumours, even those originating from the MP. ESE is a technical extension of ESD that excavates deeper into the MP. Endoscopic full-thickness resection (EFTR) requires full-thickness resection and endoscopic closure of the defect with metallic clips [9]. Submucosal tunnelling endoscopic resection (STER) utilizes a submucosal tunnel to resect the targeted tumour [10], and this technique can maintain the integrity of mucosal tissues as barriers against air or liquid leakage. A recent study demonstrated that both tunnel resection and non-tunnelling resection are safe and effective for treatment of SMTs, but tunnel resection requires a longer operation time.

Some hybrid techniques such as laparoscopic endoscopic cooperative surgery (LECS) [11], laparoscopy-assisted endoscopic full-thickness resection (LAEFTR) [12], and nonexposed endoscopic wall-inversion surgery [13] have shown promise. Additionally, robotic surgery [14] may provide the same benefit, but these above-mentioned advantages are not definite.

Multi-band mucosectomy (MBM) is well suited for the resection of large surface areas and does not require submucosal injection [15]. MBM also has the advantages of being rapid, simple, and safe for endoscopic therapy. The MBM technique has previously been used to resect granular cell tumours from the stomach and is infrequently used for oesophageal SMTs. Therefore, MBM may be useful for the removal of upper GI SMTs.

To our knowledge, no study has compared the efficacy of MBM, ESD and ESE in the setting of GI SMTs. Thus, we analysed the short-term and long-term performance of these 3 techniques for resecting upper GI SMTs in a low-volume centre.

## Methods

We retrospectively analysed our database to identify all patients with upper GI SMTs who received endoscopic therapy (excluding those who received surgery) at Tengzhou Central People’s Hospital between January 2010 and February 2015. A total of 135 patients underwent ESD (39), MBM (60) or ESE (36). The data recorded from these patients included the location, size, and histologic findings of the neoplasms, operative details, and follow-up information.

Study approval was obtained from the local ethics committee and the institutional review board of the hospital, and informed consent was obtained from all patients for data gathering and endoscopic operations. Initially, upper GI SMTs were resected using the MBM technique. ESD and ESE were introduced in March 2010. Thereafter, the treatment strategy gradually changed from preferring MBM to preferring ESD and ESE. The clinicopathological features are shown in Table 1.

### Inclusion criteria

The maximal tumour size was 30 mm, and the included tumours did not have any high-risk endoscopic ultrasound (EUS) properties (e.g., irregular margins, cystic space and heterogeneous echotexture); intraluminal SMTs lacking ulceration did not have evidence of lymph node or distant metastases. Lesions arising from the muscularis mucosa (MM) and submucosa (SM) were included. The endoscopic technique was selected based on the nature, size, and location of lesions; the endoscopist’s experience was also a primary factor in the selection of the technique used.

#### Standard ESD

Sodium hyaluronate or glycerol was injected into the SM. Then, a full circumferential incision of the mucosa was performed using a hook knife.

Next, an insulated tip (IT) knife or hook knife was used for submucosal dissection. After the procedure, the incision was closed by clipping [16].

#### ESE

This procedure is a technical extension of ESD, with deeper excavation into the MP. The major elements of ESE procedures have been widely reported elsewhere; therefore, full details will not be provided here [6, 7].

#### MBM

This technique was performed with a Duette™ MBM kit (Cook Medical, Limerick, Ireland), which allows sequential removal with no need for submucosal injection or repeated endoscope withdrawal [15, 17, 18]. In our experience, the amount of resected tissue was limited by the capacity of the apparatus (average 1.5 cm^2^). Overlapping of 10–25% between adjacent resections was allowed to prevent any remaining residue.

### Post-treatment surveillance

GI endoscopy and EUS were performed once every 3 months during the first year and then annually thereafter to detect any signs of residual or recurrent SMTs. For patients who had tumours with malignant potential, enhanced Computed tomography (CT) examinations of the neck, chest and abdomen were performed once per year to assess local lymph nodes and detect potential distant metastases.

### Histopathology

Resected tissue specimens were analysed with haematoxylin-eosin (HE) and immunohistochemical (IHC) staining. All abnormalities were classified based on guidelines established by the World Health Organization (WHO). Disagreements between the pathologists were resolved by consensus. Tumour size, positive or negative circumferential resection margins, and lymphatic or vascular invasion were all recorded and evaluated by experienced pathologists. For piecemeal resection, the specimens were reconstructed maximally.

### Outcome parameters

The primary endpoints were as follows: en bloc resection rate, endoscopic complete resection and pathologic complete resection. An en bloc resection was obtained when the entire lesion was removed in a single piece. Endoscopic complete resection was defined as the inability to identify residual tumour tissue at the resection site by endoscopy irrespective of whether en bloc resection was achieved. Pathologic complete resection was achieved when a specimen with all-negative vertical and lateral margins was obtained independent of the histological characteristics. The operation time and adverse events were evaluated as secondary endpoints. The operation time was defined as the time between the start of the operation and the withdrawal of the resected tumour, including the time required for haemostasis.

Minor bleeding was defined as requirement of any additional endoscopic haemostasis during the final endoscopic evaluation in patients who showed no evidence of clinical symptoms or abnormal laboratory test results. Massive bleeding included clinically overt bleeding that required blood transfusion or emergency surgery. The tertiary endpoint was the local recurrence rate, which was defined as a postoperative finding of any neoplastic lesion at the previous resection site.

### Statistical analysis

Statistical analysis was performed with Statistical Package for the Social Sciences version 16.0 for Windows (SPSS, Chicago, USA). Univariate analysis of quantitative data was conducted with a t test or one-way analysis of variance (ANOVA), and enumeration data were analysed with a χ2-test or Fisher’s exact test. Baseline parameters where *P*-values < 0.5 in the univariate analyses were entered into multivariate analyses. Multivariate logistic regression was conducted to identify risk factors for endoscopic resection-related complications; differences with *P*-values < 0.05 were considered significant.

## Results

### Clinical presentation in this study cohort

The baseline characteristics of our clinical cohort are shown in Table 1. No significant differences were observed among the 3 groups in sex, median age, layer of tumour origin, and histologic diagnosis. Fewer lesions < 15 mm were observed in MBM group than in the ESD an ESE groups (*P* < 0.05, Table 1).

**Table 1:** Baseline characteristics of the included patients

	ESD group (39)	MBM group (60)	ESE (36)	*P* value
Gender				0.431
Male	22 (56.4%)	38 (63.3%)	18 (50%)	
Female	17 (43.6%)	22 (36.7%)	18 (50%)	
Median age	57.6	58.2	59.1	> 0.05
Tumor site				0.584
Esophagus	16 (41.0%)	29 (48.3%)	19 (52.8%)	
Stomach	23 (58.9%)	31 (51.7%)	17 (47.2%)	
Layer of origin				0.184
MM	15 (38.5%)	28 (46.7%)	10 (27.8%)	
SM	24 (61.5%)	32 (53.3%)	26 (72.2%)	
Histologic diagnosis				0.08
GIST	14 (35.9%)	13 (21.7%)	16 (44.4%)	
Leiomyoma	23 (58.9%)	39 (65%)	19 (52.7%)	
others	2 (5.1%)	8 (13.3%)	1 (2.7%)	
Tumor size				0.001
Tumor < 15 mm	14 (35.9%)	42 (70%)	14 (38.9%)	
15 mm ≤ Tumor< 30 mm	25 (64.1%)	18 (30%)	22 (61.1%)	

No significant differences were observed in the rates of endoscopic complete resection among the three groups (*P* = 0.442, Table 2).

**Table 2:** Clinical outcomes for different endoscopic resection techniques

	ESD group (39)	MBM group (60)	ESE group (36)	*P* value
Mean resected specimen size (mm)	21.4	14.8	28.3	< 0.001
Endoscopic complete resection rate (%)	39 (100%)	59 (98.3%)	36 (100%)	0.442
En bloc resection rate (%)				
< 15 mm	13 (92.8%)	41 (97.6%)	14 (100%)	0.597
≥ 15 mm	24 (96%)	8 (44.4%)	21 (95.5%)	0.000
Pathologic complete resection rate (%)				
< 15 mm	13 (93%)	40 (95.2%)	13 (93%)	0.915
≥ 15 mm	23 (92%)	8 (44.4%)	21 (95.5%)	0.000
Procedure time (minutes)	91 ± 43	24 ± 11	101 ± 39	< 0.01
Complications (%)				
Perforation	4 (10.3%)	0 (0%)	4 (11.1%)	0.03
< 15 mm	0	0	0	–
≥ 15 mm	4	0	4	0.035
Massive bleeding	4 (10.3%)	0 (0%)	4 (11.1%)	0.03
< 15 mm	0	0	0	–
≥ 15 mm	4	0	4	0.035
Minor bleeding	3 (7.7%)	4 (6.7%)	3 (8.3%)	0.952
< 15 mm	0	1	1	0.643
≥ 15 mm	3	3	2	0.804
others	2 (5.1%)	1 (1.7%)	2 (5.5%)	0.503
Conversion to open surgery	3 (7.69%)	0 (0)	1 (2.8%)	0.057
4-year local recurrence (%)	2 (5.1%)	4 (6.7%)	1 (2.7%)	0.945

Subgroup analysis was performed based on tumour size. For lesions≥15 mm, the ESD and ESE techniques resulted in significantly higher en bloc resection rates than MBM (*P* = 0.000); no significant differences were observed for lesions < 15 mm (*P* = 0.597). The mean diameters of resected specimens were 14.8 mm for the MBM group, 21.4 mm for the ESD group, and 28.3 mm for the ESE group (*P* < 0.001).

The mean procedure time was significantly different among the three groups (*P* < 0.001), and patients in the MBM group experienced the shortest operation time (24 ± 11 min). No significant difference in operation time was observed between the ESD and ESE groups (*P* > 0.05).

The incidence of complications, such as major bleeding or perforation, also significantly differed among the three groups (*P* = 0.03). No major bleeding occurred in the MBM group; major bleeding occurred in four patients each in the ESD and ESE groups. One patient in the ESD group had a major haemorrhage that was successfully managed by haemostatic forceps and subsequent blood transfusion. One patient with massive bleeding in the ESE group was converted to open surgery. The remaining major bleeding events were successfully endoscopically treated without blood transfusion or surgical intervention.

Minor bleeding occurred in four patients in the MBM group, three patients in the ESD group, and three patients in the ESE group, resulting in no significant differences among the groups (*P* = 0.952). All minor bleeding events were treated successfully with hot biopsy forceps and argon plasma coagulation.

Perforation rates were considerably different among the 3 groups (*P* = 0.03). No perforations occurred in the MBM group. Perforation occurred in four patients each in the ESD and ESE groups while applying the coagulation current during the dissection of the SM.

Three severe perforations occurred in the ESD group, and closure of the perforations using endoclips was attempted but failed. These patients then underwent traditional thoracotomies. The remaining patients recovered after endoscopic clipping, fasting and intravenous administration of antibiotics.

In addition, conversion to open surgery did not occur for any patient in the MBM group. One case was presumptively diagnosed as a benign lesion, but the histologic diagnosis indicated a malignant lesion (1 gastric carcinoid); the patient subsequently underwent surgical resection.

### Factors associated with all the complications: univariate and multivariate analysis (Tables 3 and 4)

To reduce the potential bias as much as possible, we performed multivariate analysis. Univariate analysis showed that the complication rate was associated with tumour size, tumour origin layer, and the endoscopic procedure type (Table 3). Complication rates were higher in tumours originating from the SM [odds ratio (OR): 3.882, 95% Confidence interval (CI): 1.222–12.335, *P* = 0.022]. The ESD and ESE groups also had higher complication rates than the MBM group (OR:8.167, 95% CI: 2.436–27.379, *P* = 0.001; OR: 7.583, 95% CI 2.242–25.645, *P* = 0.001).

**Table 3:** Factors associated with all the complications: univariate analyses

Factors(n)	Univariate analysis (OR, 95%CI, P)
Gender	
Male(78)	1(reference)
Female(57)	0.985 (0.437–2.22), 0.971
Tumor site	
Esophagus (67)	1(reference)
Stomach(68)	1.78 (0.784–4.031), 0.169
Layer of origin	
MM(53)	1(reference)
SM(82)	3.882 (1.222–12.335), 0.022
Histologic diagnosis	
GIST(32)	1(reference)
Leiomyoma(92)	0.773 (0.324–1.843), 0.561
Others(11)	1.091 (0.245–4.856), 0.909
Endoscopic procedure	
MBM(60)	1(reference)
ESD(39)	8.167 (2.436–27.379), 0.001
ESE(36)	7.583 (2.242–25.645), 0.001
Tumor size	
Tumor < 15 mm(70)	1(reference)
15 mm ≤ Tumor < 30 mm(65)	1.996 (0.879–4.532), 0.098
Piecemeal resection vs En bloc resection	
Piecemeal resection	1(reference)
En bloc resection	0.493 (0.152–1.597), 0.238

**Table 4:** Factors associated with all the complications: multivariate analyses

Factors(n)	Multivariate analysis (OR, 95%CI, P)
Tumor site	
Esophagus (67)	1(reference)
Stomach(68)	1.993 (0.761–5.222), 0.16
Layer of origin	
MM(53)	1(reference)
SM(82)	2.383 (0.566–10.04), 0.237
Endoscopic procedure	
MBM(60)	1(reference)
ESD(39)	13.161 (2.529–68.502), 0.002
ESE(36)	7.542 (1.071–53.169), 0.043
Tumor size	
Tumor < 15 mm(70)	1(reference)
15 mm ≤ Tumor < 30 mm(65)	1.162 (0.424–3.182), 0.07
Piecemeal resection vs En bloc resection	
Piecemeal resection	1(reference)
En bloc resection	0.114 (0.017–0.748), 0.024

Multivariate analysis showed that only en bloc excision and the endoscopic resection techniques (ESD/ESE) were significant factors associated with complications (Table 4).

### Long-term local recurrence rates

All patients underwent follow-up for 4 years. The local recurrence rates in the MBM group were 0, 3.3, 5 and 6.7% at 1, 2, 3, and 4 years, respectively. The respective one-, two-, three- and four-year local recurrence rates were 0, 2.6, 5.1 and 5.1% in the ESD group and 0, 0, 2.7 and 2.7% in the ESE group. The overall differences in four-year local recurrence were not significant among the 3 groups (*P* = 0.945, Table 2). No deaths occurred during the follow-up period. One patient in the ESD group had local recurrence following an additional endoscopic resection procedure, and another patient underwent a laparotomy. Recurrence after MBM was treated by endotherapy in two patients and by open surgery in another two. One patient in the ESE group received open surgery.

## Discussion

No consensus is available regarding an optimal strategy for the endotherapy of SMTs. According to current guidelines, large (diameter > 3 cm) or symptomatic SMTs require surgery due to their malignant potential. However, the detection of small SMTs (diameter ≤ 3 cm) presents diagnostic and therapeutic challenges. The Canadian guidelines recommend that small GISTs (less than 1 cm) should be resected due to the risk of metastasis [19–22]. However, National Comprehensive Cancer Network (NCCN) and European Society for Medical Oncology (ESMO) guidelines recommend that lesions larger than 2 cm in diameter should be removed and biopsied. For lesions smaller than 2 cm, guidelines recommend endotherapy for oesophageal, gastric and duodenal tumours that exhibit an increase in size, change in colour, or other mucosal findings, and EUS follow-up is recommended for other sites. However, this approach requires patients to exhibit good compliance with the prescribed regimen and involves increased risk of repeated endoscopic examination and a potential risk of misdiagnosis of malignancies.

It is important to understand the mindset of the patients; some may experience stress due to the increased requirement for regular examinations and feel that it is imperative to search for a safe and efficacious form of treatment. Moreover, for many asymptomatic benign tumours, complications may develop as the tumour grows. Therefore, patients may miss the window for endoscopic treatment. Even a small SMT could be an early-stage malignant tumour. Standard endoscopic forceps biopsy and EUS-fine needle aspiration (FNA) typically fail to obtain adequate material for diagnosis of small and deep tumours [23, 24]. An accurate histopathological diagnosis can only be obtained by removing the SMT. Thus, some researchers recommended that once GISTs are suspected, they should be surgically or endoscopically removed [25, 26], though according to NCCN guidelines, immediate resection is not required [19].

Despite the popularity of STER, non-tunnelling techniques such as ESE, ESD and MBM are still widely used, especially in rural hospitals. This is the first retrospective report examining the use of the ESD, ESE and MBM techniques for treatment of GI SMTs, and the aim was to provide instructive information on their treatment.

We have shown the safety and effectiveness of MBM for resection of early oesophageal cancerous lesions [15]. MBM has been infrequently used to treat upper GI submucosal lesions; thus, it may lead to successful removal of small and superficial SMTs. In this study, we retrospectively analysed patients with SMTs limited to the submucosal layer; therefore, the STER and EFTR techniques were not included.

The complete resection rates of the MBM, ESD and ESE groups were all nearly 100%, and the differences among the 3 groups were not significant. Good performance was achieved in terms of en bloc resection and pathologic complete resection rates among all groups for lesions < 15 mm. However, for lesions≥15 mm, ESD and ESE achieved the same en bloc resection rates (100%). MBM only achieved an en bloc resection rate of 44.4%. These data imply that lesions larger than 15 mm may be better treated by ESE or ESD. Lesions smaller than 15 mm can be removed by either ESD (deep lesions) or MBM (superficial lesions). One pertinent factor might be the duration of the operation and the operative difficulty of endoscopic therapy because the average operation times of ESD and ESE are significantly longer than that of MBM, regardless of tumour size. Thus, an endoscopist might prefer MBM over ESD or ESE for superficial and small SMTs because of its low complication rate, especially for elderly and high-risk patients, for whom surgical time should be minimized. MBM cannot be performed as deeply as ESD or ESE, may not remove MP lesions, and does not always achieve a successful en bloc resection. In the present study, submucosal lesions were found in the ESD and ESE groups, and the mean resected specimen size for patients who underwent MBM was also much smaller than that in the ESD and ESE groups. The results of our retrospective study were consistent with those of other studies. Zheng et al. [27] achieved a 100% resection rate using the “band-ligate and resect” method to excise SMTs ranging from 0.5 to 5 cm in size, and no perforation or recurrence were reported. In addition, Meng et al. [28] showed that endoscopic band ligation (EBL) could markedly decrease complications, operation time, and the average length of stay compared to ESD and LAP. Regarding the long-term outcomes, both ESD and EFTR were safe and effective for removing gastric stromal tumours (< 5 cm), and no metastasis [29] was observed during the 2-year follow-up period.

To maximally decrease potential bias, multivariate analyses were conducted, which showed that the type of endoscopic procedure was a significant factor associated with complications. The incidence rates of massive haemorrhaging and perforation in the ESD and ESE groups were higher than those in the MBM group, but no significant difference was observed between the ESD and ESE groups. Although the complication rate in this study was slightly higher than that in a previous study [30], it was still within the range of 0 to 39%, revealing a high degree of variability [31, 32]. This result can be partially explained as follows. First, we have limited experience with ESD and ESE, especially for some locations and origins of lesions included in this study. Second, the total number of patients in each group differed. Third, the retrospective selection of patients may have caused overestimation or underestimation of the final results. Importantly, no deaths were associated with endoscopic therapy in this study.

Some endoscopists [33] have suggested that ESD is safe and feasible for resection of SMTs less than 8 cm in diameter. In our experience, based on this retrospective study, all 3 techniques are feasible and effective methods for GI SMTs less than 30 mm in diameter. The high pathologic complete resection rate of each endoscopic technique ensured adequate diagnosis and therapeutic strategies in all cases included. The low risk of 4-year local recurrence demonstrated the durable effects of these techniques for SMTs.

MBM had the shortest average operation time among all groups. For lesions larger than 15 mm, complete resection or en bloc resection was difficult using the MBM technique. In the present study, piecemeal resections were successful for 10 lesions larger than 15 mm, and pathologic complete resections were achieved in most patients. Piecemeal resection did not influence long-term follow-up outcomes. As demonstrated by the 4-year follow-up results, patients who were treated using the MBM technique had the same low risk of recurrence as did those who received ESD or ESE, which is a strength of this retrospective study.

This study had several limitations. First, three endoscopic resection techniques for treating different types SMT were included in this study, which might cause potential bias in the multivariate analyses. Second, this study was based on a limited number of experiences at a low-volume centre. Third, endoscopic resection was attempted in some patients based on their personal wishes; however, the study protocol specified the performance of endoscopic resection for all eligible patients. Fourth, no comparison was performed between surgical resection and endoscopic resection. Fifth, STER, EFTR and other techniques were not included.

Finally, most of the resected lesions were benign or potentially malignant, and it is unclear whether it is necessary to resect these tumours. This is controversial issue for endoscopists and surgeons, as well as in Eastern and the Western countries. Regardless, endoscopists in Western countries typically do not have the same level of expertise in ESD/ESE techniques as those in Eastern countries because of the low incidence of gastric cancer in Western countries [34]. Our study revealed that the MBM technique is safe, efficient and rapid for the treatment of GI SMTs.

## Conclusions

Taken together, the ESD and ESE techniques showed considerable advantages over MBM for en bloc resection but were associated with a higher rate of perforation and bleeding. For lesions less than 15 mm in diameter, MBM allowed for safe and easy piecemeal resection and provided the same high complete resection rates as the other techniques. MBM also had advantages such a shorter operation time and fewer complications, even in a low-volume centre. Nonetheless, all 3 techniques resulted in low recurrence rates during the 4-year follow-up. Large-scale randomized controlled clinical trials are needed in the future, especially for challenging techniques such as EFTR, STER and hybrid techniques, which require further evaluation.

## Data Availability

All data generated or analysed during this study are included in this published article and are available from the corresponding author on reasonable request.

## Abbreviations

CI: Confidence interval
CT: Computed tomography
EBL: Endoscopic band ligation
EFTR: Endoscopic full-thickness resection
ESD: Endoscopic submucosal dissection
ESE: Endoscopic submucosal excavation
ESMO: European Society for Medical Oncology
EUS: Endoscopic ultrasound
FNA: Fine needle aspiration
GI: Gastrointestinal
GISTs: Gastrointestinal stromal tumours
HE: Haematoxylin-eosin
IHC: Immunohistochemical
IT: Insulated tip
LAEFTR: Laparoscopy-assisted endoscopic full-thickness resection
LAP: Laparoscopic resectio
LECS: Laparoscopic endoscopic cooperative surgery
MBM: Multiband mucosectomy
MM: Muscularis mucosa
MP: Muscularis propria
NCCN: National Comprehensive Cancer Network
OR: Odds ratio
SM: Submucosa
SMTs: Submucosal tumours
STER: Submucosal tunnelling endoscopic resection
WHO: World Health Organization
